# Research Progress on Tofu Coagulants and Their Coagulation Mechanisms

**DOI:** 10.3390/foods13213475

**Published:** 2024-10-30

**Authors:** Yuhan Geng, Xin Du, Rui Jia, Yi Zhu, Yuhao Lu, Xiangfei Guan, Yuehan Hu, Xinyu Zhu, Minlian Zhang

**Affiliations:** 1 Department of Chemical Engineering, Institute of Biochemical Engineering, Tsinghua University, Beijing 100084, China; gyh8787@163.com (Y.G.); dux23@mails.tsinghua.edu.cn (X.D.); jiarui200108@gmail.com (R.J.); zy18801250776@163.com (Y.Z.); luyh@sioc.ac.cn (Y.L.); gxf0413@163.com (X.G.); h17803325303@163.com (Y.H.); z68547937@163.com (X.Z.); 2 School of Humanities and Social Sciences, School of Public Administration, Beihang University, Beijing 100083, China

**Keywords:** tofu, coagulant, coagulation, mechanism

## Abstract

Tofu has captivated researchers’ attention due to its distinctive texture and enrichment in nutritional elements, predominantly soybean protein. Coagulants play a critical role in promoting coagulation during tofu production, directly influencing its texture, quality, and physicochemical characteristics. Currently, the impact of coagulant characteristics on coagulation, as well as the underlying mechanisms, remain unclear. This review provides a summary of research progress on salt coagulants, acid coagulants, enzyme coagulants, novel coagulants, polysaccharide additives, and various coagulant formulations. The coagulation mechanisms of various coagulants are also discussed. Accordingly, this paper seeks to offer reliable theoretical guidance for the development of novel coagulants and the realization of fully automated tofu production.

## 1. Introduction

Tofu is a traditional Chinese food that originated during the Western Han Dynasty more than 2000 years ago. It is an important component of the East Asian diet and is becoming increasingly popular in Western countries as well. The raw material for tofu, i.e., soybean, is a high-quality source of plant protein and contains the nine essential amino acids necessary for growth and development. Additionally, soybean contains lipids, carbohydrates, crude fibers, isoflavones, minerals, and saponins, which can reduce cholesterol levels, ameliorate the symptoms of cardiovascular and kidney diseases, and reduce the incidence of cancer and tumors [1]. Digestibility studies have reported approximately 65–85% and 92–98% protein digestibility, respectively, for soymilk and bean products, such as tofu. Thus, tofu consumption could significantly improve the utilization of soybean protein [2].

Tofu production is a complex process and includes a series of operations, such as material selection, soaking, grinding, filtering, boiling, pulping, setting, and pressing. A key step in tofu production is the solidification of soymilk into tofu curd using a coagulant. This process is affected primarily by the coagulant composition and processing conditions, which directly determine the physical, chemical, and sensory characteristics of tofu. Overall, tofu yield, quality, hardness, and sensory characteristics are dependent on the type of coagulant used due to the differences in the coagulation mechanisms. The interaction between coagulant molecules or ions and soybean protein determines the quality of tofu. Moreover, the phytic acid content of soybean can significantly influence coagulant activity during tofu production [3,4].

Several studies have been performed to elucidate the mechanisms of action of different coagulants in tofu processing. To effectively summarize these studies, we have performed a thorough literature search, prioritizing studies directly related to tofu coagulants and their mechanisms of action to ensure clarity and academic credibility. We have also considered experimental design, sample size, and research outcome reliability when selecting and reviewing the most pertinent studies.

Thus, this paper seeks to examine research progress on different coagulants and their mechanisms of action in tofu processing to provide a theoretical basis for selecting coagulants for the industrial production of flavor-specific tofu. Additionally, this review serves as a reference for optimizing tofu production.

## 2. Research Progress on Different Coagulants

In ancient times, brine or gypsum was the major coagulant for tofu production. However, lactone tofu has been used in large-scale tofu production since the 1980s due to its softness, smooth texture, high water retention, and springiness. Recently, several novel coagulants have been developed based on traditional salt and acid coagulants, including enzyme, emulsion, composite, and carbohydrate auxiliary coagulants [4]. Research progress on tofu coagulants is summarized in Table 1.

### 2.1. Salt Coagulants

Salt coagulants are the oldest and most widely used tofu coagulants; these include magnesium chloride, magnesium sulfate, calcium chloride, calcium sulfate, and calcium acetate. Several metal cations have similar coagulating effects on soybean proteins.

Liu et al. [6] suggested the use of divalent cations to bind and solidify protein molecules, i.e., the “ion bridge theory”, representing one of the three major mechanisms of action of salt coagulants. Arii et al. [68] reported that the interaction between metal cations and soybean protein carboxyl groups significantly impacts tofu formation, with calcium and magnesium ions being the most significant. Meanwhile, tofu can also be produced using related metal chlorides. Therefore, based on the yield of calcium chloride tofu and other calcium salt tofu, as well as the difference in hardness between calcium sulfate, calcium gluconate, and other tofu, it can be assumed that cations are the initiators of tofu formation, while anions have greater effects on protein gelation rate and tofu retention than cations [6,68].

Different kinds of salt coagulants exert different effects on tofu properties. Lu et al. [5] reported that calcium chloride, calcium lactate, calcium acetate, and calcium gluconate can induce soybean protein coagulation at a pH of approximately 6. However, the yield and textural properties of tofu differ considerably based on the associated calcium salt. The yield of calcium chloride tofu is significantly higher than that of other calcium salt tofu, while the hardness of calcium sulfate and calcium gluconate tofu is significantly greater than that of other tofu. Murekatete et al. [69] studied the effect of the salt coagulants CaSO_4_ and MgCl_2_ on tofu coagulation, and proposed that CaSO_4_-induced coagulation produces a mass that has a more uniform network, with a finer and smoother surface finish than the mass produced during MgCl_2_-induced coagulation. Leiva et al. [70] examined the sensory properties of tofu prepared using recombinant soymilk (12% total solids) and calcium chloride at two different concentrations (0.3% and 0.5%) and found that calcium chloride improves tofu fluidity. Nguyen et al. [10] studied the use of calcium salts present in eggshell powder as an alternative coagulant in tofu preparation and observed improved textural characteristics. Prabhakaran et al. [8] further explored the effects of different coagulants on isoflavone retention in tofu, and identified calcium sulfate as the most suitable, as evidenced by high yield, high isoflavone retention ability, and superior tofu texture.

The concentration of salt coagulants plays a decisive role in determining the properties of tofu curd. Liu et al. [6] reported an increase in gel strength and a decrease in water retention with increased salt coagulant concentration. Chen et al. [15] tested the content of MgCl_2_ in 30 different types of soybean coagulants and found that the coagulant content was positively and negatively correlated with subunit content and lysine content, respectively. Hsieh et al. [11] reported that 5 mmol/L magnesium chloride coagulated the 7S subunits, 11S subunits, β-amylase, Bd 30K, sucrose-binding protein 2, lectin, and trypsin inhibitor A in soybean milk into the soymilk pellet fraction. Arii et al. [12] found that low (<10 mmol/L) and high (10–20 mmol/L) concentrations of magnesium chloride produce smooth and rough precipitates, respectively, and the precipitate quality produced with the use of high-concentration magnesium chloride decreases slightly after a long stable period. They reported an average magnesium chloride content of 10 mmol/L (range: 0–20 mmol/L) in tofu products. However, the actual tofu production process uses concentrated or saturated brine as a coagulant for soybean milk; in the initial stage of solidification, the brine can have a relatively wide concentration distribution. It is, therefore, important to expand the scope of magnesium chloride concentration in studies. Accordingly, Lu et al. [71] were the first to examine the effect of magnesium chloride concentrations (from 0 to saturation) on condensate formation, revealing that the coagulation process can be divided into five stages based on magnesium chloride concentration, i.e., no-condensation stage (0–3 mmol/L), condensation-forming stage (3–8 mmol/L), coagulation stability stage (8–50 mmol/L), condensation dissociation stage (50–210 mmol/L), and no-condensation stage (≥210 mmol/L). In the coagulation stability stage, the condensate microstructure exhibits loose and dense changes with changes in magnesium chloride concentration.

The production processes also impact tofu properties, leading many researchers to investigate means to improve the performance of tofu by altering the methods of salt coagulant addition, mixing, and heating. For example, Liu et al. [13] reported that after a one-time addition of magnesium chloride to soybean milk, the best condensation yield and sensory quality were achieved by stirring for 20–30 s at 120 rpm. Meanwhile, adding magnesium chloride in batches improves water retention, hardness, cohesiveness, and gumminess. Meanwhile, Li et al. [14] reported that alkaline heat treatment can improve the protein extraction rate; however, the use of magnesium chloride as the coagulant results in a large number of 7S α’, α subunits, and 11S acidic (A) subunits not being used upon the coagulation reaction endpoint. Zhang et al. [9] further reported that dual-frequency and multi-angle ultrasonic treatment generates high shear force, promoting exposure of the protein’s hydrophobic region and protein aggregation, resulting in better thermal stability and water retention of calcium sulfate tofu.

During the 1980s, research was focused primarily on the salt coagulant type and optimal concentration due to the belief that they critically impact the soybean milk mother stock. That is, increasing the coagulant concentration enhances the tofu coagulation strength but reduces the water-holding capacity. Meanwhile, the applicable concentration range of different salt coagulants differs; thus, for soybean milk produced by different soybean varieties, the salt coagulant concentration can be inversely deduced according to the required strength and water retention of tofu. However, recently, research has shifted to focus on discovering novel coagulants, coagulant mixtures, their optimal dosage, and their mechanisms of action.

### 2.2. Acid Coagulants

Acid coagulants predominantly use hydrogen ions to promote protein coagulation. The most widely used acid coagulant is glucono-δ-lactone, which can dissociate into gluconic acid in an aqueous solution to induce tofu curd formation. Kohyama et al. [16] plotted the coagulation curves by dynamic viscoelastic measurements and compression tests, revealing that the coagulation process can be divided into two steps: protein denaturation by heat and hydrophobic coagulation promoted by GDL. They also compared the performance of GDL with calcium salts as the coagulant, suggesting that calcium ions induce the more rapid formation of condensate. Li et al. [17] studied the effects of raw soymilk concentration and heating and solidification conditions on the water-holding capacity of lactone tofu. They found that tofu prepared using 12% raw soymilk heated at 95 °C for 20 min and solidified at 85 °C for 60 min had the best water-holding capacity.

In terms of pretreatment, certain processing technologies can alter the size of soybean milk particles, improving the texture characteristics of tofu. Yu et al. [18] pretreated soybean milk by colloid grinding, ultrasonic treatment, and homogenizer treatment, and studied the corresponding changes in the texture characteristics of tofu. Colloid grinding treatment may reduce the particle size of soybean milk so that the protein network is closer, causing increased tofu hardness. The ultrasonic treatment with higher power within a certain range can change the particle size of soybean milk, so that with increased treatment time, the hardness and springiness of tofu first increase and then decrease, whereas cohesion first increases and subsequently slowly decreases, or directly and rapidly decreases. Additionally, homogenizer treatment can cause the hardness, springiness, and cohesion of tofu to first increase and then decrease. Xu et al. [19] examined the effects of soybean soaking on the yield, protein utilization rate, hardness, and water retention of lactone tofu. When soaking soybeans with distilled water and increasing the soaking temperature, the protein extraction rate, coagulation yield, and hardness first increase and then decrease; a temperature of 25 °C is most favorable to achieve optimal protein extraction rates. Li et al. [20] compared the isothermal titration calorimetry (ITC) net heat of unheated defatted soymilk and protonation heat; the hydrophobic interaction exhibited an overt endothermic effect, increasing with soymilk temperature. When the heating temperature of soybean milk is 90 °C, the strength of the hydrophobic interaction peaks, the coagulation time is the shortest, and the gel strength is the highest.

In addition to GDL, organic acids, including lactic acid, acetic acid, succinic acid, and tartaric acid, can promote tofu solidification. For instance, Li et al. [26] produced hawthorn-flavored tofu using the natural acidic component of hawthorn as the coagulant; the quality of the end product rivaled that of commercial tofu. Meanwhile, Fasoyiro et al. [27] used the acidic nature of roselle water extract (pH 2.01–3.74) to prepare tofu. When the concentration of rose extract reached 2.5%, the tofu texture and flavor were comparable to that prepared by other natural coagulants. Song et al. [25] evaluated the suitability of several organic acids in tofu production and found that tofu produced using only lactic acid as the coagulant had the best texture properties, whereas tofu prepared using a mixture of organic acids had the best quality. Additionally, Cao et al. [28] evaluated the suitability of citric acid, malic acid, and tartaric acid as coagulants and found that the type (soft or hard) and quality (physical and chemical properties) of tofu are dependent on the acidification step during the coagulation process. Further research revealed that citric acid synergistically interacts with salt coagulants and polysaccharides to improve tofu texture and properties [65]. Zhao et al. [72] reported that adding calcium chloride and calcium sulfate to citric acid-induced tofu significantly improves the springiness and water retention of the tofu coagulation. Murekatete et al. [69] evaluated the effect of different types and concentrations of coagulants on the dynamic modulus of tofu coagulation and found that acid-induced coagulation had the highest storage modulus, whereas the gelation time decreased with increasing coagulant concentration. Meanwhile, Zeppa et al. [29] used grape pomace (GP) to prepare tofu. GP is composed of grape skins and seeds and contains high quantities of organic acids (tartaric acid, malic acid, and citric acid), calcium salts, and fiber. Organic acids and calcium salts can promote the formation of a coagulation network, while GP fibers can reduce the homogeneity of the coagulation structure. Under their combined action, the product has higher hardness and adhesiveness but lower chewiness, cohesiveness, and resilience. Khoder et al. [73] prepared tofu coagulations using different volume ratios of citric acid and nanoscale fish bones, revealing that nanoscale fish bone can serve as a functional coagulant to improve the performance of acid-induced tofu coagulation.

During tofu production, a yellow discharge containing certain nutrients is generated with the potential to contribute to environmental pollution. Wu et al. [74] proposed that the chemical oxygen demand (COD) of soybean product wastewater, predominantly yellow pulp water, markedly exceeds the standard, with an extremely high organic content that rapidly reduces dissolved oxygen in water, killing a large number of facultative anaerobes in water. However, after fermentation by lactic acid bacteria, an acid slurry containing lactic acid is obtained, which can be used as a coagulant [75], reducing the cost of coagulant and environmental pollution caused by the yellow discharge. Tofu produced using acid slurry coagulant is called acid slurry bean tofu. Liu et al. [23] examined the mechanism of acid slurry in acid slurry bean tofu production using molecular methods. The results showed an increase in the number of hydrogen ions and a decrease in the negative charge of protein molecules after acid slurry coagulant addition, which gradually reduced the surface hydrophobicity. The generation of aggregates causes the particle size distribution of the system to resemble that of large particles. Zhang et al. [24] found that increasing the acid slurry amount increases the acidification rate and gradually exposes tryptophan, tyrosine, and phenylalanine residues in the soybean milk; in this way, the hydrophobic interactions are strengthened, and the β and A3 subunits participate in initial protein aggregation, while α and α’ subunits contribute to later aggregation. With the promotion of hydrophobic interactions, protein aggregation accelerates and the coagulation network becomes finer and more compact.

Guan et al. [21] developed a novel technology utilizing pure lactic acid bacteria for fermentation to produce acid slurry bean tofu, standardizing the traditional acid slurry bean tofu production system. Zhang et al. [22] further determined that the yellow discharge from tofu production can be fermented at 42 °C for 30–35 h to obtain an acid slurry with a pH of 3.3–3.5. Tofu prepared using this coagulant at 90 °C for 20 min exhibits superior sensory characteristics. Huang et al. [76] adopted the response surface method to determine the optimal conditions for acid slurry bean tofu production, including the soybean-to-water ratio, boiling temperature, boiling time, and acid slurry content, with the tofu yield and protein content as the response parameters. The optimal conditions were as follows: soybean–water ratio of 1:5 (kg:kg), boiling time of 6.1 min, boiling temperature of 105 °C, and acid slurry content of 26%.

Given the unique flavor of acid slurry tofu, Yang et al. [77] examined the volatile flavor compounds generated during different stages of acid slurry bean tofu processing by gas chromatography–mass spectrometry and found that optimizing the boiling time and set time can induce the formation of compounds with a pleasant flavor. Wu et al. [30] investigated the physicochemical properties and metabolic products of acid slurry by adding different amounts of flour. Acid slurry rapidly produces more organic acids and amino acids with flour. Acids and ketones are the main flavor components of acid slurry, producing a unique fruity, buttery, and creamy aroma.

Presently, GDL, acid slurry, and other organic acids are used as coagulants to induce tofu coagulation production from soybean protein. Lactone tofu has rapidly become one of the main tofu varieties due to its unique taste, which differs from that of gypsum or brine tofu. However, further research is necessary to optimize the production process. Meanwhile, acid slurry bean tofu is favored because of its waste reuse characteristics. The main objectives of acid slurry bean tofu research include improving the stability of the acid slurry bean tofu quality and determining the optimal fermentation conditions. Recently, several novel organic acid coagulants have been developed, with some relatively positive research results. Chen et al. [78] proposed that the fermented yellow serous water through *Lactiplantibacillus plantarum* could be used as a coagulant to prepare yogurt tofu, which has a large water-holding capacity, large microstructure gaps, and improved tofu quality. However, most of these research findings are yet to be employed in commercial tofu production.

### 2.3. Enzyme Coagulants

In addition to salt and acid coagulants, enzyme coagulants possess great potential for tofu production as they are widely present in animal and plant tissues and microorganisms. To date, studies have been performed on pepsin, alcalase, papain, bromelain, and glutamine aminotransferase. Fuke et al. [31] confirmed the role of bromelain in the aggregation and gelation of hot soymilk by measuring thiol content and hydrophobicity. Murata et al. [79] found that tofu prepared using bromelain had higher protein recovery and yield. Luan et al. [35,80] studied 13 different proteases and found that pepsin, chymotrypsin, and flavor protease had no soymilk-coagulating ability, whereas alcalase, papain, and bromelain had strong coagulating properties.

Furthermore, microorganisms are an important source of coagulase. Yasuda et al. [34] isolated TYO-67-producing microbial soybean-milk-coagulating enzyme (SMCE) from tofu, which can produce homogeneous and smooth coagulation. Meanwhile, Murata et al. [32] evaluated the soymilk-coagulating ability of 17 commercial proteases from different sources and found that neutral or alkaline coagulase from microorganisms had a better effect on soymilk coagulation. The coagulation efficiency of soy milk and the degree of soy milk hydrolysis by microbial alkaline protease and neutral protease are higher than by microbial acid protease, bromelain, or papain animal and plant protease. Moreover, adding enzymes, calcium chloride, and magnesium chloride can increase the coagulation of soy milk protein. Further research showed that novel soybean tofu products can be obtained from enzyme-coagulated soymilk tofu through fermentation [33,81]. Yin et al. [82] found that the evaluated actinomycete nematode fermentation significantly improves the phenolic components, antioxidant activity, and nutritional components of tofu. Lo et al. [36] studied the effect of *Lactobacillus casei* FNCC-0090 and *Lactobacillus plantarum* spp. fermentation on the antioxidant capacity of tofu, and observed an improvement in radical scavenging capacity by 11.21%, with good oxidation resistance.

In 1959, Clarke et al. [83] identified TGase, which promotes acyltransferase activity in guinea pig liver. Subsequently, in the 1980s, Ikura et al. [37] reported that guinea pig liver-derived TGase can catalyze the crosslinking polymerization of soybean protein; however, its application is limited due to high purification costs. In 1989, Ando et al. [38] extracted a Ca^2+^-non-dependent TGase from *Streptomyces mobaraense*. Since the beginning of the 21st century, microbial TGase has been widely used due to its Ca^2+^-non-dependent enzyme activity, high temperature and pH stability, low cost, and high yield.

TGase can improve the coagulation properties, emulsification, and foaming properties of tofu. Xiong et al. [84] reported that the addition of TGase significantly improves the compressive capacity, viscosity, water content, water retention, and microstructure compactness of lactone tofu without significantly impacting the sensory quality. Wang et al. [85] found that the addition of calcium chloride and TGase composites significantly improves the water retention, hardness, springiness, and chewiness of tofu coagulation. Similarly, Li et al. [86] found that the addition of TGase to papain-treated soymilk increases gel strength, water retention, and unfrozen water content. Chang et al. [42] evaluated the effect of microbial TGase on the rheological and textural characteristics of black bean-packaged tofu and found that TGase increases the coagulation temperature of soymilk and improves tofu firmness and springiness, with reduced cooking loss. Nonaka et al. [39] further reported that microbial TGase pretreatment before pulping inhibits cooking-induced water loss and hardening of gypsum tofu and lactone tofu. Yang et al. [87] suggested that the capacity of TGase to improve the lactone tofu strength is predominantly related to the 7S and 11S proteins. When using TGase as the coagulant, TGase catalyzes acyl transfer reactions between intra-or inter-strand glutamine and lysine peptide residues to form covalent bonds [88]. Rui et al. [43] suggested that these covalent crosslinks will hinder the digestive enzyme hydrolysis of tofu. Furthermore, Yang et al. [44] found that combining TGase and lactic acid bacteria as coagulants improves tofu digestibility.

Processing conditions, such as enzyme quantity, treatment time, temperature, and pH, can significantly affect the crosslinking of soybean protein. Wang et al. [40,41] found that tofu hardness and gelation initially increase and then either stabilize or decrease with increasing TGase concentration, whereas the viscospringiness and cohesion are not significantly affected. Additionally, texture and rheological analysis showed that the heat treatment time of soybean milk significantly affects TGase activity. Wang et al. [89] evaluated the effect of TGase on gypsum tofu and concluded that the optimal conditions were a TGase concentration of 0.5 g/L, pulping temperature of 55 °C, and holding time of 18 min.

Alcalase, papain, and bromelain induce soybean protein tofu coagulation. However, compared with traditional inorganic salt or acid coagulants, the outcomes of these coagulants are poor, as they produce bitter-tasting curds, limiting their application. Meanwhile, TGase can improve the textural properties of tofu with potential applications in tofu production. Shi et al. [55] used TGase to prepare colorful tender tofu, characterized by better food matrix structure, gelation, and water retention, that can be combined with vegetable juice and GDL. Indeed, current research on TGase is focused on its application in other food processes, such as meat and dairy product processing. Cheng et al. [90] have shown that a certain amount of TGase treatment can induce intramolecular and intermolecular coaching, form protein molecules with larger molecular weight, and create more compact protein structures, increasing the adsorption capacity of protein to water molecules and further enhancing the hardness of egg white powder. Thus, research has begun to focus on the benefits of combining TGase with other enzymes in food processing.

### 2.4. Novel Coagulants

#### 2.4.1. Emulsion Coagulants

Research on tofu processing has shifted toward the development of novel and sustainable coagulants. Magnesium chloride has high solubility and can catalyze the solidification of soybean protein without a hydrolysis reaction. The coagulation rate of tofu curd considerably influences its characteristics. For instance, the excessively rapid coagulation rate of marinated tofu results in poor water retention, low yield, and high hardness and coarseness. However, these issues may be circumvented by altering the coagulation activity of emulsion coagulants [46,91].

The main components of emulsion coagulants include the water phase (largely comprising brine), oil phase (largely comprising natural oil), an emulsifier, and protein. Based on the diffusion mode, emulsion coagulants can be defined as oil-in-water (W/O) or water-in-oil-in-water (W/O/W) types. Zhu et al. [45] examined changes in water content during tofu processing using a differential calorimetry scanner and low-field nuclear magnetic resonance, revealing that tofu produced using a water-in-oil coagulant had higher free water content and longer relaxation time than traditional tofu. Li et al. [46,91,92,93] examined the preparation methods of W/O and W/O/W and their effects on tofu curd and found that emulsion coagulants can improve the spatial structure of tofu coagulation, reduce coagulation hardness to maintain springiness, increase water content and yield, increase the amounts of isoflavones and protein, and increase the brightness and whiteness of tofu. W/O/W coagulants also endow tofu with better dispersity than W/O coagulants [94]. Zhu et al. [95] found that with increased magnesium chloride concentration in W/O emulsions, the water content of tofu decreases, the hardness increases, and the structure becomes denser. When the concentration of magnesium chloride is 2.0 M, the tofu microstructure is the most uniform and orderly. However, when the concentration is further increased, large aggregates form and the structural uniformity is reduced [95]. Nevertheless, adding proteins, such as whey protein isolate and bovine serum albumin, into emulsion coagulants can improve the stability of the emulsions and positively affect the tofu curd quality. Zhu et al. [45] showed that adding whey protein isolate to a W/O coagulant improves the tofu water retention and quality, whereas bovine serum albumin addition retards the release of magnesium ions in W/O/W emulsions, positively influencing the microstructure of soybean protein coagulation [48]. The positive effects of whey protein isolate on the structure of W/O emulsions are due primarily to the decrease in particle size, increase in viscosity, and decrease in interfacial tension [47].

#### 2.4.2. Composite Coagulants

Gypsum, brine, GDL, and other common coagulants possess certain disadvantages, including bitter taste and residues in tofu and the slow pulping rate of gypsum. Tofu produced using brine has poor water retention and low shelf-life. Meanwhile, lactone tofu is soft and not suitable for frying [96,97,98,99].

Recently, composite coagulants using calcium salt, brine, and GDL have gained considerable research attention due to their advantages over single coagulants. Zheng [49] determined the optimal formula for a composite coagulant containing gypsum, disodium hydrogen phosphate (modifier), monoglyceride (emulsifier), and GDL, with gel strength as the evaluation index; the optimal formula was GDL 0.3%, gypsum 0.069%, disodium hydrogen phosphate 0.047%, and monoglyceride 0.019% (calculated using soybean milk). Zhang [50] determined the best concentration and ratio of a gypsum–GDL composite coagulant, with yield, water content, water-leaving rate, protein content, appearance, internal structure, and flavor as the evaluation indices, reporting that the optimal concentration of GDL and gypsum were 0.35–0.38 g and 0.18 g (2:1), respectively. Li et al. [14] found that after combining magnesium chloride and lactic acid 1:1, the coagulation presented a honeycomb structure with improved hardness, springiness, and water-holding capacity.

To rapidly determine the optimal ratio of compounds in coagulants, several researchers have adopted the principle of optimality in compound experiments, which involves selecting the concentration range with the best water content, protein content, and sensory evaluation when the coagulants act alone, and then conducting experiments according to the optimal concentration range to obtain the formulation that can produce a tofu with the best texture. In this way, Wang et al. [51] determined that the best GDL, calcium acetate, and magnesium chloride ratio for optimal gel strength, water retention, and sensory characteristics is 2:1:1. Similarly, Shi et al. [52] reported that the best gypsum and brine ratio for optimal tofu strength, chewiness, and sensory characteristics was 4:6. Additionally, Wang et al. [53] found that the optimal gypsum, magnesium chloride, and GDL ratio was 5:3:2, based on yield, water content, water retention, protein content, and sensory evaluation.

Recently, the addition of enzymes has diversified coagulant formulation. Shi et al. [55] applied TGase and GDL to colored tender tofu and studied the proportion of compound coagulant and pulping temperature using the response surface method. The optimal conditions obtained for the springiness and color of tofu included TGase and GDL concentrations of 1.4 g/L and 1.7 g/L, respectively, and pulping temperature of 53 °C. Yang et al. [44] examined the coagulation properties and digestibility of tofu prepared using a composite coagulant comprising TGase and lactic acid bacteria fermentation liquid, and found that the addition of 4 U/g TGase significantly improved the water retention, chewiness, and hardness of tofu. Additionally, the structure of tofu processed using 2 U/g of enzyme was beneficial for digestion, providing a theoretical basis for tofu digestion. Yue et al. [56] determined that the optimal ratio of calcium sulfate, magnesium chloride, GDL, and TGase in the coagulant formulation to achieve optimal gel strength was 0.5% CaSO_4_ + 0.3% TGase, 0.4% MgCl_2_ + 0.3% TGase, and 0.4% GDL + 0.1% TGase. Additionally, tofu prepared using CaSO_4_ + TGase had superior nutritional and sensory characteristics than that prepared using the other combinations. Xie et al. [100] reported that the optimal coagulant composition to prepare whole soybean tofu coagulant comprised 17% soybean fermentation broth, 0.2% CaSO_4_, and 0.2% TGase. Overall, these studies expand the application range of soybean fermentation broth and provide theoretical support for the processing and application of whole soybean products. Gao et al. [58] found that combining arabinoxylan and soymilk protein with peroxidase improves the effect of TGase on tofu performance. The optimal conditions for water retention, hardness, storage modulus, and loss modulus of tofu are 1.0% arabinoxylan + 100 U peroxidase/g arabinoxylan + 1 mL 3% H_2_O_2_/g arabinoxylan + 25 U TGase/g protein. Lu et al. [101] proposed a novel method for whole soybean flour tofu preparation by combining calcium sulfate and GDL coagulation. When the calcium sulfate–GDL ratio is 3:2, the crosslinked network gel structure can be improved, with superior water content and flavor.

The types of composite coagulants and their optimal formulations are listed in Table 2. All tofu coagulants can be used to formulate composite coagulants, demonstrating the huge advantages and development potential of composite coagulants. However, the gelation mechanism of composite coagulants remains unclear, limiting their application.

#### 2.4.3. Carbohydrate Auxiliary Agents

In addition to the use of multiple coagulants, some studies have examined the effect of additives, such as carbohydrates, in tofu production. The addition of carbohydrate as an auxiliary agent in the soymilk coagulation process can effectively improve tofu characteristics [63].

Li et al. [63] compared the effects of complex (magnesium chloride and guar gum complex) and traditional (gypsum tofu and brine tofu) coagulants, reporting that guar gum affects the coagulation structure and texture characteristics of tofu by altering the solidification rate of soymilk. Cao et al. [65] evaluated the synergistic effects of coagulants and polysaccharides and found that they significantly improve the water retention, hardness, springiness, and hydrophobic interaction of tofu. Li et al. [54] evaluated the effects of adding sodium chloride and starch to composite coagulants, such as GDL and brine, identifying the optimal composite coagulants by measuring the tofu gel strength, water-leaving rate, and sensory characteristics. Zhao et al. [66] examined the effects of adding polysaccharides, such as konjac, gellan, and kodeland, to a calcium sulfate-induced soy protein coagulation on texture properties. Polysaccharide addition enhanced the coagulation structure, accelerated gelation, improved the coagulation microstructure, and reduced the coagulation onset temperature.

Additionally, researchers have evaluated the suitability of chitosan acetic acid solution as a novel coagulant for tofu [57,60]. Zhao et al. [61] added chitosan to improve the water retention and shelf life of pressurized lactone tofu. Jun et al. [62] used crab shell extract treated with acetic acid as a coagulant; the properties of the prepared tofu were equivalent to those of brine tofu and lactone tofu. No et al. [59] reported that tofu ash prepared using chitosan had low protein content, good sensory quality, long shelf life, and high commercial value. Additionally, Yu et al. [57] determined the concentration of chitosan and acetic acid coagulants for optimal gel strength, dehydration rate, and sensory characteristics. Tofu prepared using 1.0% chitosan, 1.2% acetic acid solution, 90 °C pulping temperature, and 30 min set time had optimal physicochemical and sensory characteristics.

Li et al. [102] investigated the effect of conjugates between soybean isolate proteins and dextran on the quality of GDL tofu. The addition of soybean isolate protein dextran conjugates impacts the gel strength, water retention, microstructure, and color of tofu. Conjugates containing more hydroxyl groups are effective in improving the water retention of tofu. Zhang et al. [67] studied the influence of glycinin–dextran conjugate on the texture characteristics and micromorphology of TGase tofu. The yield, hardness, and gel strength of tofu were increased, and the microstructure was more compact.

## 3. Research Progress on Coagulation Mechanism

### 3.1. Effect of Phytic Acid on the Coagulation Process

Phytic acid (C_6_H_18_O_24_P_6_), also known as creatine or inositol hexaphosphate, is an organophosphate derived from plant seeds. It is widely found in several plant-derived foods, with the highest content in legume seeds and bran and germ grains [103]. The addition of phytic acid or phytate to soymilk has an obvious effect on the texture characteristics of tofu.

Studies on the effect of phytic acid on the tofu condensation process date back to 1992. Schaefer et al. [104] argued that phytic acid can preferentially bind calcium coagulant, influencing the yield, composition, texture, and microstructure of tofu. Tsumura et al. [105] treated soymilk with phytase to reduce the plant acid content and found that the phytase-treated tofu had higher fracture stress than the untreated tofu. Toda et al. [106] examined the relationship between phytic acid and the protein content and fracture stress of tofu, revealing that the phytic acid content of soybean was more influenced than the protein content by environmental factors. Moreover, the fracture stress of tofu was significantly negatively associated with the phytic acid content of soymilk, indicating that phytic acid has a greater effect on tofu quality. Additionally, increased MgCl_2_ concentration causes the correlation between phytic acid content and the tofu breaking stress of tofu to decrease. To clarify the effect of phytic acid on bean tofu curd, Ishiguro et al. [107] measured the phytate content of 27 soybeans and found that softer tofu texture is associated with higher phytic acid content. These findings further indicate that the phytic acid content of soybean was more influenced than the protein content by environmental factors and that tofu quality is affected by phytic acid content. Further experimentation [108], using calcium chloride as the coagulant and sodium hydroxide, showed a decrease in hardness and an increase in viscosity and brittleness of tofu at the same coagulant concentration, confirming the impact of phytic acid content on the texture and quality of tofu. Huang et al. [109] observed that tofu produced from soybeans with high phytic acid content was difficult to shape and had poor taste. To ensure high-quality tofu, soybean phytic acid content should be maintained between 17 and 20 mg/g. Zhang et al. [110] noted that soybean phytic acid content was positively related to tofu cohesion, hardness, water retention, and water content. Ibrahim et al. [111] showed that the seed-soaking temperature can affect phytic acid content. By using three independent variables (coagulant type, coagulant concentration, and seed-soaking temperature), the three-fold bidirectional interaction significantly affects the yield and hardness of tofu, while the phytic acid content in tofu decreases, which has a corresponding impact on tofu quality.

Wang et al. [112] prepared tofu using low-phytic-acid soymilk produced by ultrafiltration, with gypsum as a coagulant. They found that the removal of free small molecules reduces the activation energy of protein condensation, accelerates the reaction, and improves gel strength, but causes a decrease in the energy storage modulus. Wang et al. [113,114] evaluated the effect of phytic acid on the tofu solidification process, and found that phytic acid forms a complex with calcium/magnesium and soybean proteins. The reaction of phytic acid and soybean protein can inhibit the thermal aggregation of soybean protein; however, phytic acid preferentially conducts reversible binding to calcium when calcium coagulant is added, reducing the direct reaction between calcium and protein, allowing the coagulation phase to produce a “buffer period.”

Given that the phytic acid content of soybean is more influenced than the protein content by environmental factors, regulation of tofu quality is challenging. Overall, in the process of coagulation, the higher the phytic acid content, the lower the tofu hardness, and the higher the viscosity and fracture stress. Table 3 summarizes the effects of phytic acid on the properties of tofu with different coagulants.

### 3.2. Coagulation Mechanisms of Different Coagulants

Heating is an important step in tofu production that can affect the properties of the tofu curd. Liu et al. [7] analyzed the thermal denaturation temperature of soybean protein by differential scanning calorimetry and found that the denaturing temperature of large bean globulin (92 °C) is ~20 °C higher than that of β-conglycinin (71 °C). Further studies showed selective denaturation of soybean protein by two-step heating at 75 °C for 5 min and 95 °C for 5 min, which increased the springiness of tofu curd compared with one-step heating (95 °C for 5 min) [7]. The heat generated by the food during the ohmic heating is correlated with the electrical conductivity. Li et al. [115] developed an experimental device for ohmic heating to control the gelation temperature, heating rate, and time-measure impedance of tofu and introduced an electrical impedance method to characterize the gelation process of soymilk. Zhang et al. [116] determined the appropriate amount of calcium sulfate for tofu production and the pulp temperature of home-made tofu. Liu et al. [117] resolved the reaction dynamics of the lactone-induced gelation process, revealing a different coagulation mechanism between heating temperatures < 70 °C and >70 °C. When the temperature is <70 °C, protein crosslinking requires high enthalpy contribution and low entropy changes, so the gelation rate is slow and will form a relatively soft curd. When the temperature is >70 °C, the reaction groups are exposed, the enthalpy contribution required for gelation decreases and the entropy required increases, thus increasing the gelation rate that will form a rough condensate.

Regarding the coagulation process, Fuke et al. [31] hypothesized that 11S globulin may be critical to gelation, while 7S protein has an inhibitory role. This study determined the surface hydrophobic and sulfhydryl content of soy-isolated proteins and speculated that disulfide bonds and hydrophobic effects are the main causes of the gelation process. Zhong et al. [118,119,120,121] studied the viscoelastic motility during the coagulation process of soybean protein, and found that 11S protein is the predominant factor in low-coagulant-concentration conditions, while 7S protein has a more important role under high-concentration conditions; hydrophobic and hydrogen bonding are the major intermolecular forces regulating the coagulation process of soybean protein.

Several metal salts can promote soybean protein coagulation. Traditional studies suggest that the coagulation process mechanism can be divided into two steps [16]: (1) thermal denaturation of proteins and (2) hydrophobic coagulation promoted by metal ions or acid protons. Arii et al. [68] suggested that the metal ions in salt coagulants act as the initiator of coagulation, and the interaction between the metal ions and the carboxyl group of aspartic acid and glutamate side chains induces curd formation. Ono [122] et al. suggested that metal ions in salt coagulants are neutralized, allowing protein particles around the oil body to coagulate. Using optical microscopy and SEM, Lee et al. [123] found that isoelectric point precipitation and calcium coagulation do not impact the spheroid structure of soybean proteins, whereas heating can disrupt the protein structure. Thermal denaturation of the protein is necessary to form an aggregated network structure. The 3D network structure of the aggregates generated by heated soybean protein boasts a low sedimentation rate, high coagulation rate, high water retention, low hardness, and high springiness. Hu et al. [124] evaluated the effect of physical force on the coagulation texture of soybean proteins and found that neutral salt at low concentrations neutralizes the protein surface charge, whereas anions under a high salt concentration exert a complex effect on soybean protein. Duan et al. [125] examined the influence of intermolecular forces on the soybean protein coagulation formation process and texture characteristics and found that electrostatic interactions, hydrophobic interactions, and hydrogen bonding had important effects on the formation of the soybean protein coagulation. Furthermore, Yang et al. [126] divided soybean protein coagulation formation into three processes: denaturation of soybean protein, aggregation of protein molecular chains, and connection of protein molecular chains. Among these, aggregation of the protein molecular chains is affected primarily by electrostatic and hydrophobic interactions, whereas connecting protein molecular chains is predominantly impacted by the hydrogen bonds and disulfide bonds. Zhou et al. [127] reported that electrostatic interactions, disulfide bonding, hydrophobic interactions, and hydrogen bonding are important in forming the tofu coagulation. Furthermore, Jin et al. [128] examined the influence of gelation temperature on the intermolecular forces in tofu and found a rapid increase in the filled tofu coagulation elastic modulus (G’) and viscosity modulus (G’), a significant decrease in the proportion of ionic and hydrogen bonds, and an increase in the proportion of hydrophobic interactions and disulfide bonds with increases in gelation temperature. While the cation in the salt coagulant is the initiator of tofu formation, anions contribute to protein denaturation or dissolution [12], which has a greater effect on the protein gelation rate and tofu retention than the cation [6,68]. However, when the mechanism of condensation is discussed or schematic diagrams of the coagulation process are constructed, the initiating effect of cations is highlighted with minimal emphasis on the effects of anions. This leads to incomplete derivations of the associated mechanisms of salt coagulants.

The coagulation process induced by GDL is similar to that of a salt coagulant, with the coagulation curve consistent with the primary reaction kinetics; however, GDL induces a lower solidification speed than a salt coagulant [121]. Liu et al. [129] evaluated the effects of different coagulants on intermolecular forces in tofu curd formation and found that both the hydrophobic effect and disulfide bonds play important roles in soybean coagulation formation, with both markedly higher in gypsum tofu than in lactone tofu. Cavallieri et al. [130] studied the coagulation behavior of whey protein isolate solution under condensing conditions with GDL as the coagulant agent. By analyzing the rheological energy of the system under initial coagulation and local rearrangement, the gelation process was divided into three steps: coagulation core formation, crosslinking gel strengthening, and uniaxial compression. Ju et al. [131] found that GDL reduced the pH of the system and induced the basic polypeptide and β subunit to form the initial condensed network, followed by the α, α’subunit and acidic polypeptide supplement to the condensed structure, forming a condensed network with greater hardness and finer structure.

Furthermore, adding enzymes, sugar, and other substances to soymilk can affect the coagulation process. Polysaccharides regulate milk coagulation by influencing the solidification rate of soybean protein. Li et al. [63] reported that guar gum has different effects on the two stages of soymilk solidification, accelerating protein aggregation in the first stage and slowing the formation of protein supramolecular structural units of coagulation networks in the second phase. Additionally, under the action of guar gum, the supramolecular structural unit of soybean protein is tighter, whereas the overall network structure is loose.

The effect of TGase on tofu coagulation properties is due primarily to its interaction with soy protein. Wang et al. [40,41] showed that TGase catalyzes the formation of covalent bonds during tofu coagulation, promoting and reinforcing the coagulation network structure. Sakamoto et al. [132] found that the number of covalent bonds increased with TGase. Hence, TGase may promote milk coagulation by catalyzing the crosslinking reaction. However, Yasir et al. [133] compared tofu made of ordinary soybean milk treated with MTGase, and found that although the coagulation network obtained after enzymatic treatment was more delicate and uniform, the electrophoresis results showed almost no crosslinking. Therefore, Masir posited that, in the formation of the coagulation network, the crosslinking reaction of TGase makes negligible contributions to coagulation; instead, side reactions (such as hydrolysis of glutamine residues) likely play important roles. Nevertheless, Yang et al. [87] showed that the α’, α subunit in 7S and the A_3_ peptide chain in 11S are most closely related to TGase, followed by the β and γ subunits in 7S and the A peptide chain in 11S. Analysis of the amino acid content of these subunits and peptide chains showed that TGase is closely related to lysine in soy proteins.

**Figure 1:**
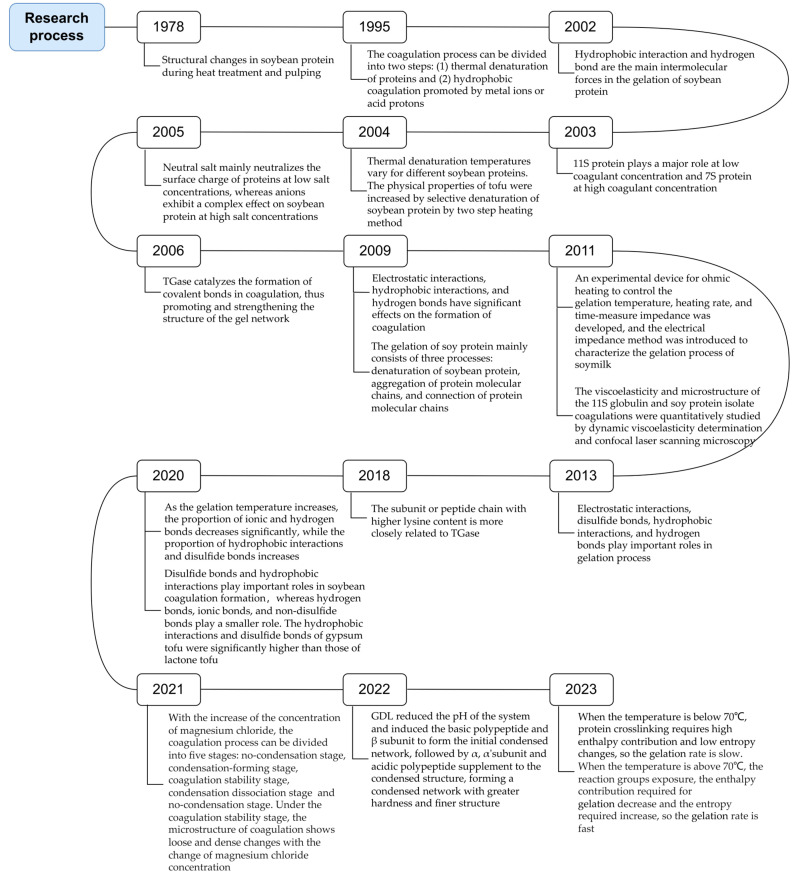
Research progress related to the tofu coagulation mechanism [7,16,40,41,71,87,115,117,118,119,120,121,123,124,125,126,127,128,129,131,136].

**Figure 2:**
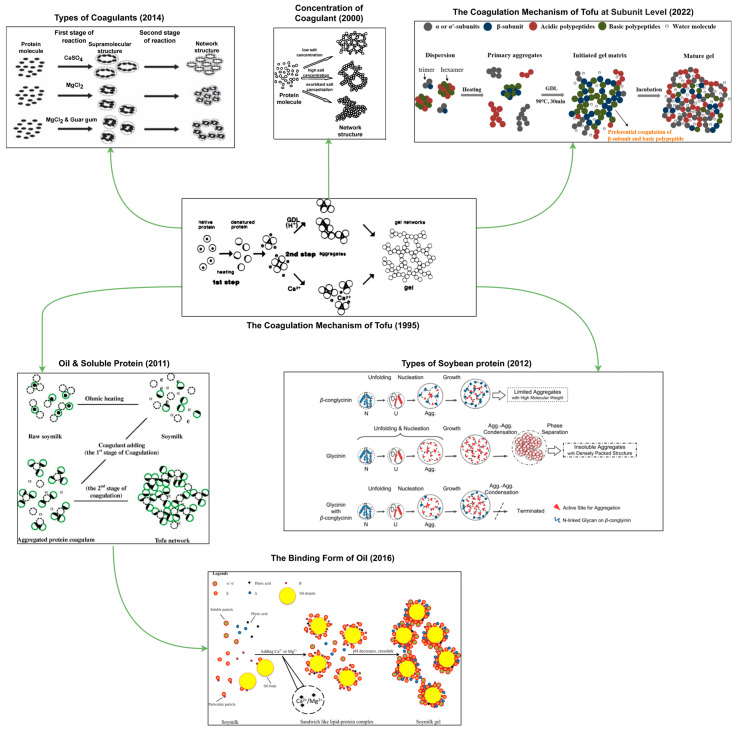
Schematic diagram of the tofu coagulation mechanism [6,16,63,115,131,134,137].

Figure 1 shows the research progress in studying the tofu coagulation mechanism from the 1970s to the present. Figure 2 is a schematic representation of the tofu coagulation mechanism. In 1978, the ion bridge theory was proposed, advancing tofu research toward the elucidation of coagulation mechanisms [123]. In 1995, Kohyama et al. [16] confirmed from the perspective of dynamics that the coagulation process induced by calcium ions and GDL is divided into two stages, creating a schematic of the coagulation mechanism. This schematic was subsequently referenced to modify and expand a series of studies on the mechanism of the coagulation process [134]. Moreover, different types of soybean proteins associated with the curdling mechanism were identified and further explored to establish the mechanism of curdling. Tang et al. [135] then proposed a sandwich model for the curdling mechanism by advancing from the protein level to the lipid and protein sublevels. Cumulatively, these studies have provided detailed discussions regarding the mechanisms of coagulation from the perspectives of coagulant type, concentration, protein type, protein composition, interaction, etc. However, the associated outcomes were inferred based on indirect experimental data, with no direct observational data used to verify their accuracy. In addition, although the soybean composition is complex, it has been simplified in current studies that investigate the mechanism of tofu curd, deviating from reality. Thus, efforts are underway to experimentally design simulations of the real curdling process.

## 4. Conclusions and Research Prospects

This paper summarizes the research progress of salt coagulants, acid coagulants, enzyme coagulants, new coagulants, polysaccharide additives, and other coagulants, and discusses the associated coagulation mechanisms. The study of compound coagulants helps improve the disadvantages of single coagulants in terms of flavor, nutrition, and taste. Emulsion-type coagulants improve the spatial structure through the controllable release of coagulants. Moreover, the use of polysaccharides as coagulation aids can enhance the gel structure and accelerate the gelation process. An increase in phytic acid—a component of soybean—decreases the hardness of tofu while increasing the viscosity and fracture stress. Meanwhile, salt coagulants are kinetically similar to the coagulation process induced by GDL; generally, the coagulation process promotes hydrophobic clotting by metal ions or acidic protons after the thermal denaturation of proteins. Four physical forces—electrostatic interactions, disulfide bonds, hydrophobic interactions, and hydrogen bonding—are important in the gel-formation process. TGase likely promotes coagulation by (1) promoting curd formation by catalyzing the crosslinking reaction or (2) by participating in side reactions, such as glutamine residue hydrolysis, causing isoelectric point changes that promote coagulation formation.

Presently, research on tofu coagulants is focused on salt, acid, and enzyme coagulants. Recently, researchers have explored the factors affecting the quality of tofu and developed novel coagulants, providing a theoretical basis for producing tofu. Although novel coagulants, such as natural organic, protease, and emulsion coagulants, are yet to be commercialized for tofu production, they possess considerable potential for improving tofu quality. Research on coagulants is also markedly impacted by regional characteristics and industrial development. For example, waste grape residue from the brewing industry has been investigated as a tofu coagulant in terms of its performance [24], contributing to green production and reducing the cost of by-product treatment. Moreover, the processes (e.g., production components, pretreatment, heating methods, coagulant formula) for industrially producing tofu are designed to produce characteristics (e.g., hardness, elasticity, gel strength, water retention, and flavor) that will achieve optimal consumerism. Research that elucidates effective strategies and methodologies to create optimal coagulants helps inform and maximize the efficient industrial production of tofu while minimizing the trial-and-error process of each factory. Indeed, in the current social environment, pursuing a low-carbon and healthy diet is becoming increasingly common. Hence, tofu, as a food with high nutritional value, low-calorie density, and environmental friendliness, has increased in popularity, making its efficient production an important research and industrial focus.

However, studies have yet to fully examine certain aspects of the mechanism of tofu coagulants. Presently, studies on tofu curd are focused on the macro physical and chemical properties of tofu, with few evaluating the micro-curd mechanism. Additionally, most research outcomes have not been applied in commercial tofu production. Therefore, further studies are necessary to explore the micro mechanisms of tofu and the development of novel coagulants. Additionally, further research, development, and policies are necessary to facilitate the commercialization of research findings for sustainable tofu production. In-depth discussions of the coagulation mechanism of tofu help guide the scientific production of tofu in terms of comprehensive composition, structure and performance, and soybean breeding. Meanwhile, tofu, as a food gel, has good renewable, biodegradable, and edible properties. Hence, an in-depth understanding of its coagulation mechanism and structure is conducive to developing innovative food gels with high nutritional value, as fat substitutes, and for targeted delivery, among other applications.

## Figures and Tables

**Table 1:** Research progress on tofu coagulants.

Type	Type	Year	Research Results
Salt	Calcium salts	1980	Calcium salts and two non-calcium compounds induce coagulation production of soybean protein [5]
		2000	Effects of calcium salt coagulants on gel strength and water retention of tofu [6]
		2004	Thermal denaturation temperature of soybean protein [7]
		2006	Effects of different coagulants on the retention of isoflavones in tofu [8]
		2022	Effects of dual-frequency and multi-angle ultrasound on the network structure and texture properties of calcium sulfate tofu [9]
		2023	Influence of chicken eggshell powder as an alternative coagulant on the yield and textural characteristics of tofu [10]
	Magnesium salts	2000	Effects of magnesium chloride on tofu gel strength and water retention [6]
		2012	Proteomic analysis of tofu formation induced by magnesium chloride [11]
		2013	Effects of magnesium chloride concentration on tofu properties [12]
		2017	Effects of mixing speed, mixing time, and magnesium chloride adding batch in the pulping stage on the tofu yield [13]
		2023	Effects of alkaline heat treatment on texture properties of magnesium chloride tofu [14]
		2024	Mathematical modeling of optimal coagulant dosage for tofu preparation using MgCl_2_ [15]
Acid	GDL	1995	Dynamic viscoelastic measurements and compression tests infer the gelation of the lactone tofu [16]
		2003	Effects of raw soymilk concentration, heating conditions, and solidification conditions on water retention of lactone tofu [17]
		2014	Effects of pretreatment methods of soymilk on texture characteristics of lactone tofu [18]
		2022	Effects of soybean soaking conditions on the yield, protein utilization rate, hardness, and water retention of lactone tofu [19]
		2024	Effects of soybean milk heating conditions on the formation of coagulation [20]
	Acid slurry	2009	Production of acid slurry bean tofu using pure lactic acid bacteria fermentation [21]
		2014	Optimum conditions for preparing tofu using sour pulp [22]
		2019	Mechanism of acid slurry bean tofu and response of soybean protein to heating and pulping [23]
		2022	Effects of the added acid slurry amount on protein subunit aggregation [24]
	Organic acid	2012	Coagulating properties of various organic acids [25]
		2012	Tofu preparation using natural hawthorn extract as coagulant [26]
		2014	Tofu preparation using roselle water extract as coagulant [27]
		2017	Tofu preparation using citric acid, malic acid, and tartaric acid [28]
		2021	Tofu preparation using grape pomace as coagulant [29]
		2023	Effect of flour addition on the physicochemical and metabolome in acid slurry [30]
Enzyme	Coagulase	1985	Role of bromelain in aggregation and gelation of hot soymilk [31]
		1987	Neutral or alkaline coagulase from microorganisms had a better effect on the coagulation of soymilk [32]
		1989	Enzyme coagulated soymilk can be fermented to obtain new soybean tofu products [33]
		1999	Serine protease from *Bacillus pumilus* TYO-67 had an effect on the coagulation of soymilk [34]
		2006	Pepsin, chymosin, and flavor protease had no soya bean milk-coagulating activity, whereas alcalase, papain, and bromelain had strong coagulating activity [35]
		2022	Effects of *Lactobacillus casei* FNCC-0090 and Lactobacillus plantarum spp. fermentation on the antioxidant capacity of tofu [36]
	TGase	1980	Glutamine aminotransferase from guinea pig liver can catalyze the crosslinking polymerization of soybean protein [37]
		1989	Extraction of Ca^2+^ non-dependent glutamine aminotransferase from Streptomyces [38]
		1996	Addition of microbial TGase to gypsum tofu and lactone tofu [39]
		2006	TGase affects tofu hardness and gelation, but not springiness or cohesion [40,41]
		2010	Addition of TGase increases the coagulation temperature of soymilk, and improved the firmness and springiness of tofu [42]
		2016	TGase forms strong inter/intra molecular bonds between soy proteins, hindering digestive enzymatic hydrolysis [43]
		2021	Combining TGase and lactic acid bacteria can improve the digestibility of tofu [44]
Novel	Emulsion type	2014	Changes in moisture content during the oil–water–brine tofu coagulation formation process [45]
		2014	Method of preparation of two emulsion coagulants (W/O, W/O/W) and their effect on tofu coagulation [46]
		2015	Addition of whey protein isolate (WPI) to W/O coagulant improves water retention and quality of tofu [47]
		2017	Effect of W/O emulsion on tofu texture properties and microstructure [48]
	Reassortment (composition)	2000	GDL (main), gypsum, disodium hydrogen phosphate (modifier), monovinegar (emulsifier) [49]
		2002	GDL, gypsum [50]
		2006	GDL, calcium acetate, magnesium chloride [51]
		2007	Gypsum, brine [52]
		2010	GDL, gypsum, magnesium chloride [53]
		2018	GDL, magnesium chloride hexahydrate, sodium chloride, starch [54]
		2019	GDL, TGase [55]
		2020	Calcium sulfate, magnesium chloride and GDL respectively compounds with TGase [56]
		2020	Chitosan, acetic acid [57]
		2024	Arabinoxylan, H_2_O_2_, peroxidase, and TGase [58]
	Carbohydrate (chitosan)	2010	Tofu ash prepared from chitosan has low protein content, good sensory quality, and long shelf life [59]
		2012	Preparation of tofu using chitosan and acetic acid complex as coagulant [60]
		2012	Addition of chitosan to pressurized lactone tofu improves water retention and increases shelf life [61]
		2019	Crab shell extract treated with acetic acid as coagulant [62]
	Carbohydrate (other polysaccharides)	2014	Magnesium chloride and guar gum were compounded; guar gum modified soymilk solidification rate [63]
		2015	Three polysaccharides: carrageenan, guar gum, and gum Arabic, complexed with MgCl [64]
		2018	Citric acid, salt, and polysaccharide compounding significantly improves tofu texture [65]
		2020	Konjac, gellan, and kodeland added to calcium sulfate induces soy sepharose system [66]
		2021	Glycinin–dextran conjugate and TGase compounding improve the coagulation texture characteristics and microscopic morphology [67]

Notes: GDL: glucono-δ-lacton; TGase: transglutaminase.

**Table 2:** Composition and optimal ratio of compound coagulants.

Composition	Evaluation Index	Optimum Ratio and Conditions
GDL, gypsum, disodium hydrogen phosphate (modifier), monoglyceride (emulsifier) [49]	Gel strength	GDL 0.3%, gypsum 0.069%, disodium hydrogen phosphate 0.047%, monoglyceride 0.019% (calculated by soymilk)
GDL, gypsum [50]	Yield, water content, protein content, sensory characteristics	Ratio of GDL and gypsum is 2:1
GDL, calcium acetate, magnesium chloride [51]	Gel strength, water retention, sensory characteristics	Optimal ratio of GDL, calcium acetate, magnesium chloride is 2:1:1
Gypsum, brine [52]	Strength, chewiness, sensory characteristics	Ratio of gypsum and brine is 4:6
GDL, gypsum, magnesium chloride [53]	Yield, water content, water retention, protein content, sensory characteristics	Ratio of GDL, gypsum, magnesium chloride is 2:1:1
GDL, magnesium chloride hexahydrate, sodium chloride, starch [54]	Gel strength, water-leaving rate, sensory characteristics	Ratio of coagulants (mass ratio of GDL and magnesium chloride hexahydrate) is 0.2:0.15, 0.1 mol/L NaCl 0.6 mL, 0.2% starch 3 mL
GDL, TGase [55]	springiness, coherence	Concentration of GDL is 1.7 g/L and TGase is 1.4 g/L, the temperature of pulping is 53 °C
Calcium sulfate, magnesium chloride and GDL respectively compounds with TGase [56]	Gel strength, the contents of amino acids, fats and so on	0.4% GDL + 0.1% TGase, 0.5%CaSO4 + 0.3% TGase (prepared tofu with highest quality), 0.4% MgCl_2_ + 0.3% TGase
Chitosan, acetic acid [57]	Gel strength, dehydration rate, sensory characteristics	Concentrations of chitosan is 1.0% and acetic acid is 1.2%, pulping temperature is 90 °C, set time is 30 min
Gypsum, soybean fermentation broth, TGase [100]	Water retention, springiness	Addition amount of soybean fermentation broth is 17%, CaSO_4_ is 0.2%, and TGase is 0.2‰
Arabinoxylan, H_2_O_2_, peroxidase and TGase [58]	Water retention, hardness, storage modulus, loss modulus	1.0% Arabinoxylan + 100 U peroxidase/g Arabinoxylan + 1 mL 3% H_2_O_2_/g Arabinoxylan + 25 U TGase/g protein

**Table 3:** Effect of phytic acid on the coagulation effect of different coagulants.

Coagulant	Research Contents	Results
GDL	Effect of phytic acid on the quality of GDL tofu	Phytase-treated GDL tofu has higher fracture stress than untreated tofu [105]
MgCl_2_	Relationship between phytic acid, protein content, and tofu fracture stress	Soybean phytic acid content is more influenced than protein content by environmental factors; tofu fracture stress is negatively associated with phytic acid content; the association between fracture stress and phytic acid content decreases with an increase in magnesium chloride concentration [106]
CaSO_4_/GDL	Phytic acid content and its influence on tofu quality	Tofu with higher phytic acid content has a softer texture [107]
CaCl2	Effect of phytic acid concentration on hardness and viscosity of tofu curd	The condensation hardness, viscosity, and fragility of tofu decreases with an increase in phytic acid concentration [108]
CaSO_4_	Binding form of phytic acid in soybean milk and its effect on the coagulation rate	Phytic acid slows the protein coagulation reaction, affecting gel strength [112,113]

## Data Availability

No new data were created or analyzed in this study. Data sharing is not applicable to this article.
